# Association Between Vitamin D Deficiency and Adverse Events Following Shoulder Surgeries: A Systematic Review and Meta-analysis

**DOI:** 10.1007/s43465-025-01424-3

**Published:** 2025-06-04

**Authors:** Dimitrios V. Papadopoulos, Athanasios Kontogiannis, Nikolaos Stavropoulos, Vasileios N. Nikolaou, George C. Babis

**Affiliations:** Second Department of Orthopaedics, School of Medicine, National and Kapodistrian University of Athens, Athens, Greece

**Keywords:** Vitamin D deficiency, Shoulder surgery complications, Revision surgery, Rotator cuff repair, Total shoulder arthroplasty

## Abstract

**Background and Objectives:**

The impact of Vitamin D deficiency on postoperative outcomes following orthopaedic surgeries has been a topic of great interest over the past decade. We aimed to investigate whether vitamin D deficiency is associated with an increased rate of adverse events following rotator cuff repairs (RCR) and total shoulder arthroplasties (TSA).

**Materials and Methods:**

PUBMED and SCOPUS databases were searched from February to May 2023 for studies investigating the association between vitamin D deficiency and adverse events following RCR and TSA. Studies were eligible for inclusion if they: (1) were observational cohort studies or case–control studies, (2) included preoperative Vitamin D levels or ICD-9/ICD-10 codes appropriate for Vitamin D deficiency, (3) provided an effect estimate with its Odds Ratio (OR) and 95% confidence intervals (CI), (4) were available in English. Adverse events included rotator cuff retears following RCR, and revision surgeries for any reason following RCR or TSA. Publication bias was assessed using Begg’s and Egger’s tests. Data from eligible studies was extracted and pooled OR with 95% CI were calculated using STATA version 15.

**Results:**

Four publications were included, reporting 4 independent cohort studies with > 41,000 subjects. Vitamin D deficient patients were 1.2 times more likely to experience adverse events following RCR or TSA than those without Vitamin D deficiency (OR: 1.23, 95% CI: 1.14–1.34, *p* < 0.001). The subgroup analysis of studies evaluating only RCRs revealed that the likelihood of adverse events following RCR was higher in vitamin D deficient patients than those without vitamin D deficiency (OR: 1.19, 95% CI: 1.09–1.29, *p* = 0.019).

**Conclusions:**

Vitamin D deficiency accounts for higher rates of adverse events following RCR and TSA. However, further research is required to identify the underlying pathophysiology involved in this association between Vitamin D deficiency and adverse events following shoulder surgeries.

**Level of evidence:**

IV.

## Introduction

The estimated prevalence of Vitamin D deficiency is up to 32% in the US, while one billion people worldwide are deemed insufficient [1]. Vitamin D insufficiency/deficiency is especially common among orthopaedic patients as preoperative evaluation of patients undergoing trauma surgeries or joint replacements often reveals low hydroxy-vitamin D (25-OHD) levels. Adequate Vitamin D is essential for osseous structural integrity since it maintains calcium circulation, promotes bone mineralization, and regulates bone remodelling through osteoblast and osteoclast activation [1–3]. However, over the past years, special attention has been paid to several other effects of Vitamin D including its antimicrobial properties, immunoregulative action, and its impact on wound healing [1, 4, 5].

Hypovitaminosis D has been associated with problematic wound healing, suboptimal tissue repair, and increased infection rate [1, 4, 5]. In line with this, vitamin D deficiency can affect the healing rate and functional outcomes in various orthopaedic surgeries. Especially in shoulder surgeries, vitamin D deficiency can be associated with certain complications such as retears following rotator cuff repairs (RCR) due to impaired healing potential. Moreover, periprosthetic fractures and implant loosening following total shoulder arthroplasties (TSA) can be more common in patients with Vitamin D deficiency due to decreased bone strength and altered bone ingrowth, resulting in a higher revision rate. It is estimated that more than 250,000 RCR and 40,000 TSA are performed annually in the US, accounting for three to five billion dollars in medical costs each year [6]. Given the potential implications of Vitamin D deficiency on the postoperative outcomes of these procedures, a significant burden on patient’s quality of life and the public health system can be linked to vitamin D deficiency [6–8]. That being the case, no studies have associated vitamin D deficiency with complication rates following shoulder surgeries.

The purpose of this study was to investigate whether vitamin D deficiency is associated with a higher rate of adverse events following rotator cuff repairs and shoulder arthroplasties through a comprehensive meta-analysis of the currently available literature.

## Materials and Methods

This systematic review and meta-analysis was conducted following the PRISMA 2020 guidelines [9]. The research protocol of this meta-analysis is approved by the International Prospective Register of Systematic Reviews (PROSPERO: http://www.crd.york.ac.uk/prospero) with register number CRD42023459168. No alterations to the initial protocol were made. Available from: https://www.crd.york.ac.uk/prospero/display_record.php?ID=CRD42023459168.

### Identification and Selection of Studies

A systematic review of the electronic databases PUBMED and SCOPUS was conducted from February to May 2023 to identify studies that assessed the association between vitamin D deficiency and the rate of adverse events following shoulder surgeries. The keywords that were used in the initial search algorithm, were relevant to the type of operation (“shoulder surgery”, “shoulder replacement”, “shoulder arthroplasty”, “rotator cuff”), the exposure (“vitamin D”), and the potential outcomes/complications (“infection”, “outcome”, complication”). Two reviewers (AK and DVP) identified relevant studies through an examination of the titles and abstracts of the available publications. Irrelevant studies were omitted at this stage, and the full context of relevant articles was then thoroughly evaluated to determine whether they met the inclusion criteria of this meta-analysis. Finally, the references included in the relevant articles were also evaluated to identify any other eligible studies.

### Study Eligibility Criteria

Studies were considered eligible for inclusion when they met the following criteria: (1) eligible studies included observational cohort studies and case–control studies, focusing on the association between vitamin D deficiency and adverse events in patients undergoing shoulder surgery (rotator cuff repairs and shoulder arthroplasties); (2) the studies included the evaluation of Vitamin D levels (serum 25-OHD), either preoperatively or through ICD-9 or ICD-10 codes -in the case of hospital-based and publication-based studies, respectively; (3) the studies provided an effect estimate with its odds ratio (OR) and confidence intervals (CI) or sufficient data to calculate a crude effect estimate; (4) studies were available in English. Publications that did not meet the inclusion criteria were omitted. When population overlap was observed, only the most recent or most comprehensive (i.e., with the highest number of cases) was included. Adverse events in this study included rotator cuff retears following repairs, and revision surgeries for any reason following rotator cuff repairs or shoulder arthroplasties.

None of the relevant studies evaluated Vitamin D insufficiency in correlation with postoperative complications and therefore no study was excluded for that reason. There is no consensus in the definition of Vitamin D deficiency and insufficiency. Vitamin D insufficiency and deficiency are defined as serum 25OHD levels lower than 30 ng/ml and 20 ng/ml, respectively [10, 11]. One population-based study used ICD-9 and ICD-10 codes to identify diagnoses of Vitamin D deficiency [12], while the other either an ICD-9/ICD-10 diagnosis or 25OHD levels lower than 32 ng/ml [13]. In contrast, the two hospital-based studies used different thresholds to identify Vitamin D deficiency (i.e., 20 ng/ml [14] and 30 ng/ml [15]).

### Data Collection and Quality Assessment

The included studies were reviewed in detail and the following variables were extracted: author’s details and publication year, study design, time period, setting (hospital or database study), type of surgery (RCR or shoulder arthroplasty), exposure (vitamin D deficiency), reported outcomes, number of subjects and revision cases, OR and 95% CI along with method of control for potential confounders. When adjusted estimates of effect were unavailable, crude estimates were calculated.

The risk of bias for the included studies was assessed through the Newcastle–Ottawa Scale (NOS) [16], a validated scale that evaluates information on three predefined fields: selection of the evaluated study groups, comparability of these groups, and ascertainment of either the outcome of interest (i.e., rate of adverse events) or exposure (i.e., vitamin D deficiency) for cohort or case–control studies, respectively. Each study could be attributed with four, two and three stars, for selection, comparability, and outcome or exposure assessment respectively. Therefore, the highest score could be nine stars for each study, while less than six stars indicate a high risk of bias for a study.

### Quantitative Data Synthesis

Data synthesis involved calculating the pooled effect estimates and their 95% Confidence Intervals (CI) using a random-effects model (DerSimonian–Laird approach) [17]. Moreover, publication bias was evaluated using Begg’s and Egger’s tests while Cochran’s test with a 0.10 significance level tested the heterogeneity of the included studies [18–20]. In addition, the I-squared statistic was calculated to assess the variability of the results of the included studies, which occur due to heterogeneity among the studies, rather than chance; I-squared values were categorized as follows: > 75% indicating considerable heterogeneity, 60–75% substantial heterogeneity, 30–59% moderate and < 30% low heterogeneity [21, 22]. Last, subgroup analyses were conducted, to evaluate the heterogeneity of the included studies due to study setting (population-based vs. hospital-based studies) or because of the type of shoulder surgery (TSA vs RCR).

The STATA software, version 15, was utilized for statistical analysis, while statistical significance was set at *p* < 0.05 for all the tests, except for heterogeneity. The results of the meta-analysis are presented in tables and forest plots. 

## Results

### Search Results

**Fig. 1:**
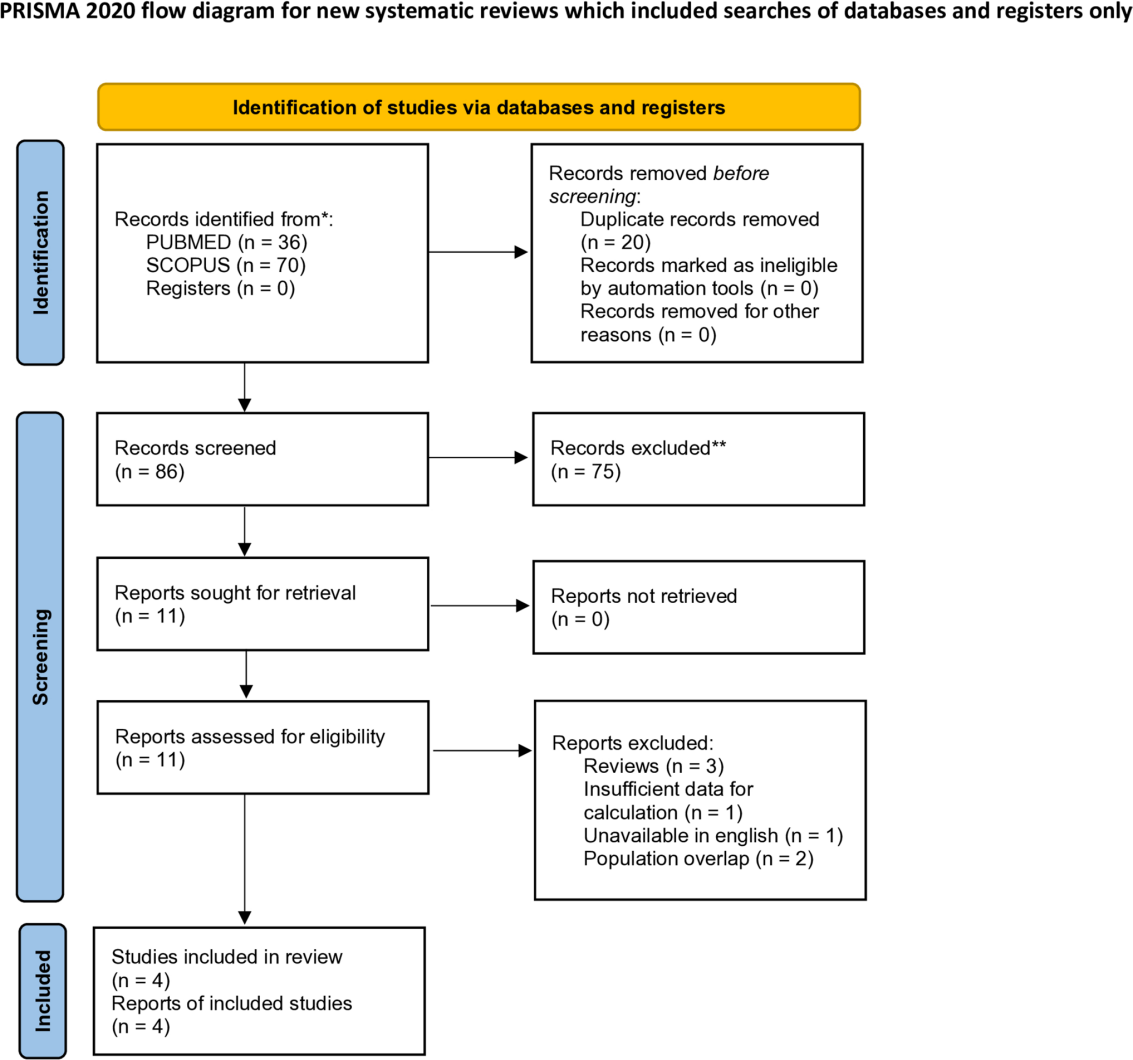
PRISMA 2020 flow diagram for new systematic reviews which includes searches of databases and registers only

As shown in the flowchart, after the initial database search and the removal of duplicate articles, our search yielded 86 citations (Fig. 1). Studies irrelevant to the topic of interest were omitted, after the screening of titles and abstracts, and therefore 11 articles remained for detailed examination through full-text review. A thorough examination of the references of the included studies was additionally performed, for potentially relevant studies. Two studies [23, 24] were excluded due to population overlap with one included study [12]. The latter presented the highest number of participants and was therefore included. Finally, the meta-analysis included 4 studies that met all inclusion criteria. Of those, an evidence level of III was attributed to 2 cohort studies and one case–control study [13–15], while one study was a case series study and yielded an evidence level of IV [12].

**Fig. 2:**
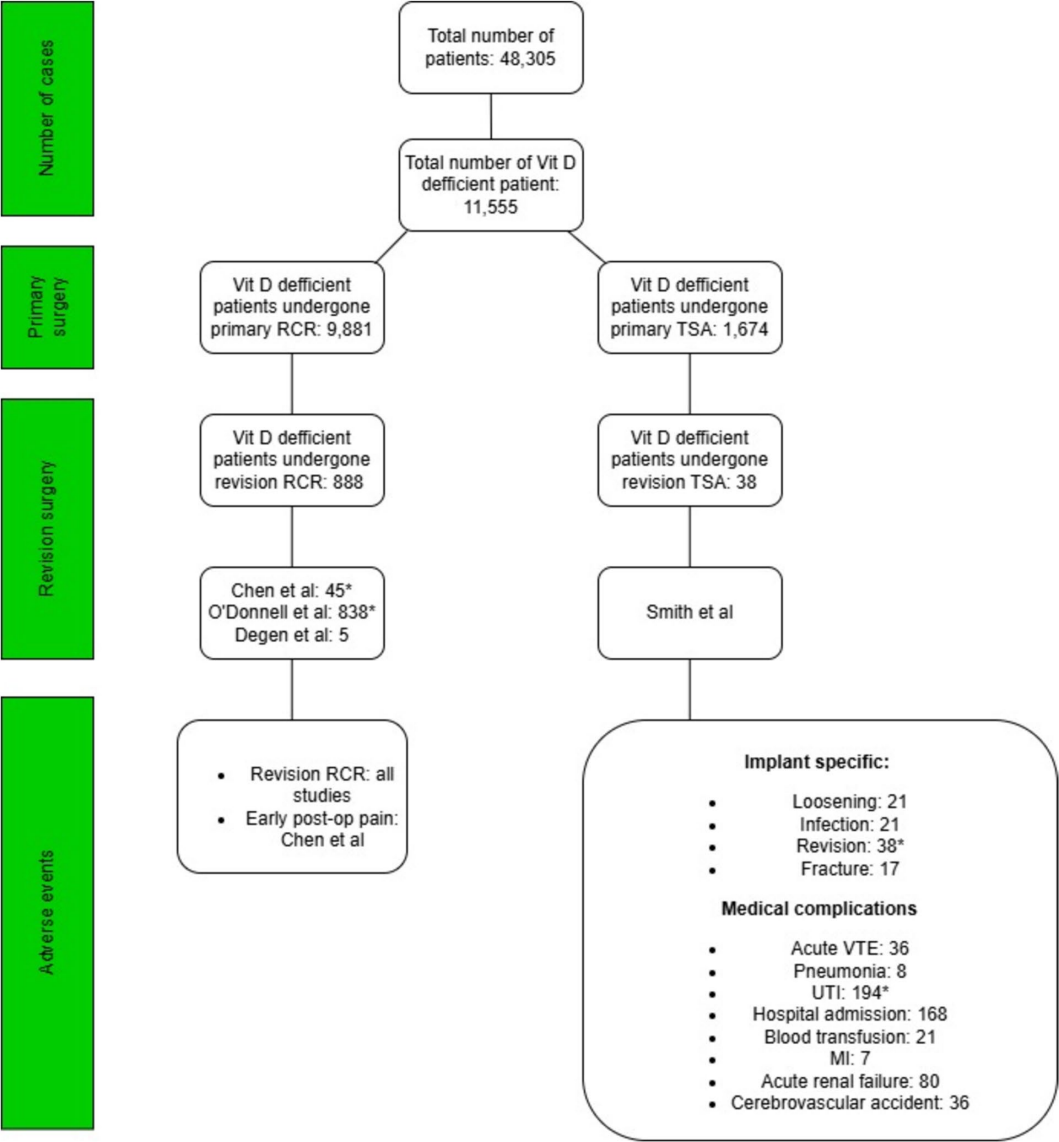
Total number of adverse events

Three of the included studies examined vitamin D deficiency as a risk factor for adverse events following RCR [12, 14, 15] two of which evaluated rotator cuff retears as adverse events. In contrast, one evaluated revision surgeries as adverse events [14]. The fourth study examined the correlation between vitamin D deficiency and TSA revision [13]. The effect estimate reported (or provided sufficient data to calculate) in the included studies was the Odds Ratio (OR) with its 95% CI. Three studies were retrospective cohort studies [12–14] while one was prospective [15]. Moreover, two of the included studies were based on hospital settings [14, 15] and the other two were population-based studies [12, 13]. Regarding the type of surgery, the number of exposed cases, and adverse events, the results were as follows: The overall number of primary RCRs included in this meta-analysis was 41,609 cases, and 9,881 vitamin D deficient patients underwent RCR. The number of revision surgeries following RCR in Vitamin D deficient patients ranged from 5 to 838, totalling 855 cases [12, 14, 15]. The number of primary TSA cases included in this study was 6,696, while there were 1,674 Vitamin D deficient patients undergoing TSA. The number of revision surgeries following TSA in Vitamin D deficient patients was 38 TSA revisions [13] The total number of adverse events cases are summarized in Fig. 2. Confounding factors were adjusted in one of the studies evaluating the risk for RCR revision and the one assessing the risk for TSA revision [12, 13]; both studies were population-based. The two hospital-based studies assessing the risk for retear following RCR revision were not adjusted for any confounder [14, 15]. Regarding the time period, the studies were published between 2016 and 2022 and only one was conducted outside of the USA (China) [14]. Study characteristics are presented in Table 1.

**Table 1:** Studies included in the meta-analysis

Study, publication year	Country	Time period	Design	Setting	Surgery	Exposure	Outcomes	No. of subjects	‘Adverse event’ cases	OR	(95% CI)	Control for potential confounding factors^a^
Degen et al., 2016 [15]	USA	2007–2016	Cohort	Hospital	RCR	Vitamin D deficiency	Rotator cuff retear	53	5	1.05	(0.03–2.84)	Unadjusted
Smith et al., 2020 [13]	USA	2007–2016	Cohort	Pearldiver Database	Shoulder arthroplasty	Vitamin D deficiency	Revision surgery	6696	38	3.32	(2.17–5.09)	Adjusted: 1–4, 6,7
O’Donnel et al., 2020 [12]	USA	2007–2016	Cohort	Pearldiver Database	RCR	Vitamin D deficiency	Revision RCR	41,467	3072	1.18	(1.08–1.28)	Adjusted: 1–6
Chen et al., 2022 [14]	China	2018–2019	Cohort	Hospital	RCR	Vitamin D deficiency	Rotator cuff retear	89	12	3.63	(1.07–12.34)	Unadjusted

CI, confidence interval; NR, not reported; OR, odds ratio; RCR, rotator cuff repair

^a^1,age; 2,sex; 3,BMI; 4, smoking status; 5,race; 6,diabetes mellitus; 7,CCI χ^2^

### Results of Meta-analysis

**Fig. 3:**
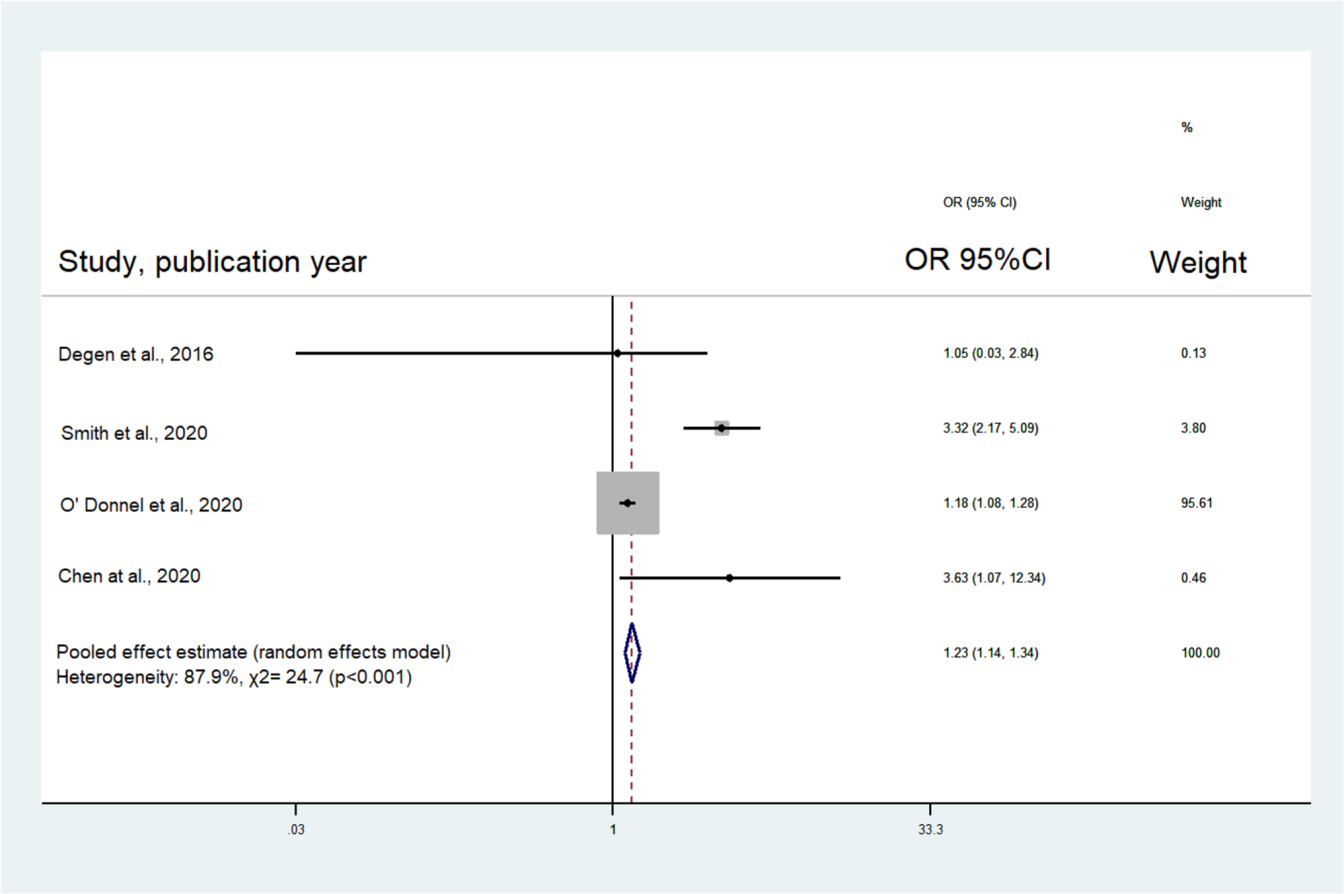
Forest plot for revision surgery after shoulder surgery (RCR and TSA) in Vitamin D-deficient patients. OR, odds ratio; CI, confidence intervals; RCR, rotator cuff repair; TSA, total shoulder arthroplasty

**Fig. 4:**
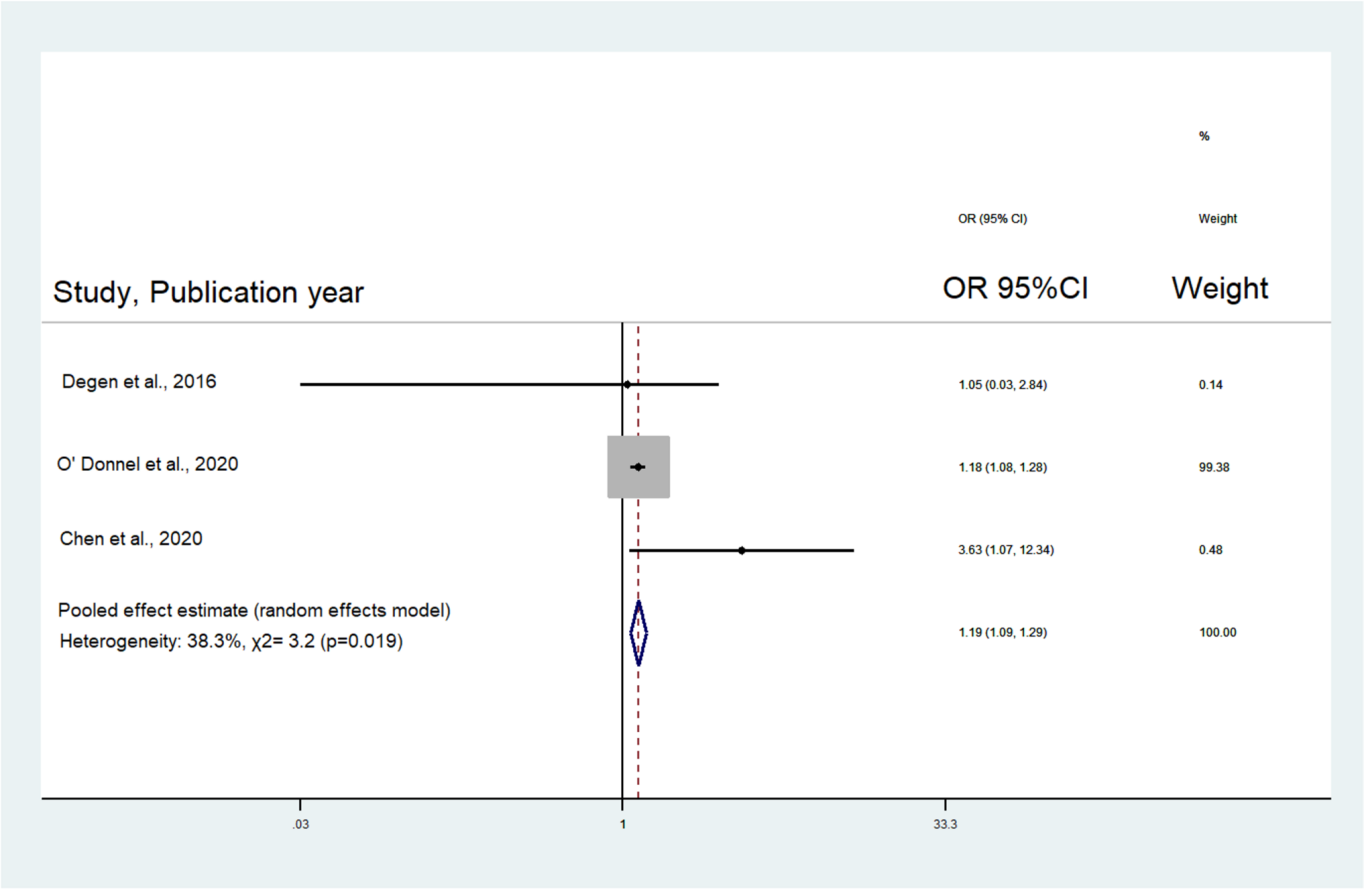
Forest plot for revision surgery after RCR in Vitamin D-deficient patients. OR, odds ratio; CI, confidence intervals; RCR, rotator cuff repair

Analysis of the 4 included studies concluded that the risk of adverse events following shoulder surgeries was 1.2 times higher in vitamin D deficient patients compared to those without vitamin D deficiency (OR: 1.23, 95% CI: 1.14–1.34, *p* < 0.001; Table 2**, **Fig. 3). Specifically, the subgroup analysis of studies evaluating only RCRs revealed that the likelihood of adverse events following RCR was also higher in vitamin D deficient patients compared to those without vitamin D deficiency (OR: 1.19, 95% CI: 1.09–1.29, *p* < 0.019 Table 2**, **Fig. 4). The estimated *p*-values for the Begg’s and Egger’s tests were 0.30 and 0.25 for all the revision surgeries and 0.99 and 0.55 for RCR revisions. As such, publication bias is unlikely. However, the I-squared values were estimated at 87.9% and 38.3% for all shoulder surgeries and RCR revisions respectively, suggesting moderate to substantial heterogenicity (Table 2).

**Table 2:** Meta-analysis results

	No. of studies	Random-effects model		Tests of homogeneity			Tests of publication bias	
		OR	(95% CI)	Q value (d.f.)	*p*-value	*I*^2^	Begg’s *p*-value	Egger’s *p*-value
Revision surgeries- All studies	4	1.23	(1.14–1.34)	24.78 (3)	< 0.001	87.9%	0.30	0.25
Revision surgeries- Rotator cuff repairs	3	1.19	(1.09–1.29)	3.24 (2)	0.019	38.3%	0.99	0.55
Revision surgeries- Hospital setting	2	2.74	(0.93–8.07)	0.89 (1)	0.34	0.0%	–	–
Revision surgeries- Database setting	2	1.22	(1.12–1.33)	21.76 (1)	< 0.001	95.4%	–	–

OR, odds ratio; CI, confidence interval; d.f., degrees of freedom

Further analysis by study setting revealed that vitamin D deficiency was associated with adverse events in database studies (OR: 1.22 95% CI: 1.12–1.33, *p* < 0.001), however, vitamin D deficiency was not associated with adverse events in hospital-based studies (OR: 2.74, 95% CI: 0.93–8.07, *p* = 0.89), as presented in Table 2.

### Risk of bias

The Newcastle–Ottawa scale was utilized to evaluate the included studies for potential risk of bias. A total of 9 stars was accredited to two studies [12, 13] while the other two received seven stars [14] [15]. All four studies had ≥ seven stars and were classified as high-quality studies according to the Newcastle–Ottawa scale (Table 3).

**Table 3:** Risk-of-bias assessment for the studies included in the meta-analysis

Study	Item 1	Item 2	Item 3	Item 4	Item 5 A	Item 5B	Item 6	Item 7	Item 8
Degen et al. 2016 [15]									
Smith et al. 2020 [13]									
O’Donnel et al. 2020 [12]									
Chen et al. 2022 [14]									

Items in cohort studies: 1, representativeness of exposed cohort; 2, selection of the non-exposed cohort; 3, ascertainment of exposure; 4, demonstration that outcome of interest was not present at start of study; 5 A, comparability of cohorts (on the basis of the design or analysis) regarding age; 5B, comparability of cohorts regarding disease extent and disease duration; 6, assessment of outcome; 7, follow-up was long enough for outcomes to occur; 8, adequacy of follow-up of cohorts

The Newcastle–Ottawa Scale (NOS), a validated tool that incorprates information on three predefined domains: selection of the study groups; comparability of the groups; and ascertainment of either the exposure or outcome of interest for case–control or cohort studies, respectively. Studies could get a total of four stars for selection, two for comparability, and three for assessment of the outcome or exposure for a total of nine stars per study. Studies scoring at least six stars were considered as of low risk of bias (i.e., high quality)

## Discussion

There has been intensive research regarding the role of vitamin D deficiency on postoperative outcomes following orthopaedic surgeries, indicating that hypovitaminosis D is associated with various surgical complications such as wound healing problems and infections [3, 25–27]. This systematic review and meta-analysis focuses on the association between perioperative vitamin D deficiency and postoperative complications in shoulder surgeries. Despite the high number of studies regarding the role of vitamin D in orthopaedic surgeries, this is the first and most comprehensive systematic review to examine the effect of hypovitaminosis D on shoulder surgeries. Our theory that Vitamin D deficiency is an independent risk factor for adverse events in RCR and TSA is confirmed. Specifically, patients with Vitamin D deficiency are 1.2 times more likely to have a retear or revision surgery [12–15] following RCR and TSA compared to those without vitamin D deficiency.

The association between higher rates of adverse events and vitamin D deficiency following rotator cuff repairs is due to several reasons. Most importantly, muscular fatty degeneration is associated with hypovitaminosis D [28]. Therefore, the positive association between hypovitaminosis D and higher revision rate following RCR that was seen in our study could be due to a higher retear rate, resulting from a more prominent fatty degeneration of the rotator cuff muscles in vitamin D deficient patients. Moreover, the higher revision rate in vitamin D deficient patients following RCR could be due to the impaired healing potential of the repaired tendons. To support this theory, experimental studies on animals have shown that vitamin D increases the number of type 1 collagen fibres and decreases type 3 collagen fibres, making the healed tendon similar to its original structure [29]. Conversely, vitamin D deficiency is correlated with poor collagen organisation on the repaired rotator cuff tendons [29]. Additionally, matrix metalloproteases (MMP) also play a vital role in tendon repair, while their excessive expression—induced by inflammation—has been associated with degenerative tendinopathy of the rotator cuff [30]. Reportedly, calcitriol, the active form of vitamin D, mitigates MMP production and therefore tendon regeneration. It is hypothesized that vitamin D deficient patients may experience impaired tendon healing, due to lack of MMP downregulation [31].

A recent study by Mouli et al. compared the effectiveness of two vitamin D supplementation strategies for patients scheduled to undergo knee arthroplasty. Evidently, a weekly loading dose of oral D3 50,000 IU improved 25-OHD levels to normal in 73.3% of the cases, compared to a daily regimen of 1,000 to 6,000 IU (42.4% *p* < 0.001) [32]. However, no association between supplementation therapy and surgical outcomes was presented. Nevertheless, a similar clinical trial could be conducted, highlighting the effectiveness of supplementation therapy before and the surgical outcomes after RCR and TSA. To further strengthen this argument, Patel et al. and Arshi et al. predicted the cost-effectiveness of vitamin D repletion before arthroscopic RCR and total knee arthroplasty, respectively [33, 34]. Specifically, supplementation therapy results in mean-cost savings of over 11 million dollars per 250,000 primary RCRs [33].

Regarding the adverse events, those included RC retears, TSA revision and medical complications [12–15]. O’Donnel et al. found that increased revision rate following RCR is associated with several parameters such as male gender, age 60–69, smoking, obesity, hyperlipidemia, and Vitamin D deficiency [12]. The most prevalent comorbidities were hyperlipidemia, Diabetes Mellitus, obesity and Vitamin D deficiency [12]. Although this is the largest included study, the nature of the population-based setting and the ICD-9 coding may underestimate the number of RCR revisions or the need for revision surgery in the general population and the studied timeframe [12]. Interestingly, Chen et al. reported a strong correlation between vitamin D deficiency and higher VAS scores at 1 and 3 months postoperatively, which could be explained by the lack of anti-inflammatory response of the cells which is normally promoted by vitamin D [14, 35]. The authors of this study also reported an association between Vitamin D deficiency and fatty degeneration of the supraspinatus, which is in line with our hypothesis about impaired tendon healing in deficient patients [14]. Vitamin D supplementation treatment could therefore accelerate tendon healing and minimise postoperative pain. Patient demographics, symptom duration—median: 2 (1–7.5) months—and comorbidities did not differ significantly between the two groups [14]. Regarding TSA, Smith et al. found a strong association between Vitamin D deficiency and all-cause TSA revision, while no association was observed between Vitamin D deficiency and specific complications such as implant loosening, lysis, or periprosthetic fracture. Authors attributed this fact to the restricted number of patients and reported that larger studies could clarify the relationship between these specific adverse events and vitamin D levels [13]. Additionally, significant differences were observed in patient characteristics (i.e., African American race) and comorbidities (i.e., morbid obesity, Diabetes Mellitus, hyperlipidemia, hypertension and tobacco use, kidney and liver disease, depression and osteoporosis) which were controlled for in the regression analysis [13]. Regarding medical complications, a significantly higher frequency of urinary tract infections was observed in Vitamin D-deficient patients [13]. The latter could be attributed to the immunoregulatory role of Vitamin D. Lastly, Degen et al.'s study was the only one that reported no association between rotator cuff retears and vitamin D deficiency. This could be due to the small population of the study since this is the smallest included study in this meta-analysis [15]. In the case of hospital-based studies, the retears were identified by ultrasound imaging at 6 months [15] and MRI imaging at 24 months [14] after surgery. O'Donnell et al., also reported a median time between primary and revision RCR of 214 days [12].

This study is limited by several factors. Most noticeably, the relatively low number of studies in the analysis could be considered its primary weakness. However, due to the high number of total cases and the high quality of the included studies (Table 3) our study does not lose validity, concerning the results of overall revision surgeries, RCR, and TSA revisions. Another limitation of this study is the high heterogeneity among the included studies, for instance, results differed between hospital-based and population-based studies. Although low 25-OHD levels were associated with a higher revision rate in population-based studies, this was not observed in hospital-based studies. This fact draws largely on the lower number of participants, and therefore revision cases, in the hospital-based, compared to population-based studies, that may prevent an association between 25-OHD levels and revision surgery. Therefore, the high difference in population number, the study setting (hospital vs database), the lack of consensus in defining the threshold for Vitamin D deficiency and the lack of adjustment for confounding factors in the two hospital-based studies [14, 15], contribute to high heterogeneity between the studies. More studies are, therefore, required to confirm our results in the hospital setting.

## Conclusion

The results of this study indicate that Vitamin D deficiency is a modifiable risk factor for adverse events following shoulder surgeries since Vitamin D-deficient patients are 1.2 times more likely to experience adverse events following RCR and TSA. Although the exact reason for the higher rate of adverse events in Vitamin D deficient patients revealed in our study is not clear, the findings of this study indicate that physicians should consider preoperative evaluation of Vitamin D levels as part of the routine screening in patients undergoing shoulder surgeries. New research opportunities considering supplementation therapy are also suggested. 

## Data Availability

Data are available upon request.
